# Summary of the National Advisory Committee on Immunization (NACI) Statement—Updated guidance on Imvamune in the context of a routine immunization program

**DOI:** 10.14745/ccdr.v51i01a01

**Published:** 2025-01-02

**Authors:** Nicole Forbes, Josh Montroy, Marina I Salvadori, Kristin Klein

**Affiliations:** 1 Centre for Immunization and Respiratory Infectious Diseases, Public Health Agency of Canada, Ottawa, ON; 2 Department of Pediatrics, McGill University, Montréal, QC; 3 Department of Medicine, University of Alberta, Edmonton, AB

**Keywords:** National Advisory Committee on Immunization, mpox, Canada, Imvamune, vaccine guidance

## Abstract

**Background:**

Mpox is a viral illness related to smallpox. It can cause flu-like symptoms and a rash, and in severe cases, can lead to hospitalization or death. The Imvamune® vaccine offers protection against mpox. Consistent with global trends, mpox cases in Canada have been reported primarily among men who have sex with men (MSM), with sexual contact as the predominantly reported mode of transmission. While the incidence of mpox in Canada has significantly declined since the fall of 2022, mpox remains an important public health concern with the potential for future resurgence.

**Methods:**

The National Advisory Committee on Immunization (NACI) reviewed available evidence on the clinical benefits and risks of Imvamune. This evidence included studies assessing the vaccine effectiveness estimates from real-world evidence, as well as pre- and post-market licensure safety data. NACI has also considered additional factors including ethics, equity, feasibility and acceptability. Guidance on the use of Imvamune in the context of international travel was developed in collaboration with the Canadian Committee to Advise on Tropical Medicine and Travel (CATMAT).

**Results:**

NACI concluded that available evidence supported the vaccine’s effectiveness and safety in preventing mpox infection.

**Conclusion:**

Building on previous interim guidance from NACI recommending the use of Imvamune for pre-exposure vaccination in the context of ongoing mpox outbreaks, NACI now recommends that Imvamune be used in the context of a focused routine immunization program. Individuals at high risk of mpox, including MSM who meet high-risk criteria such as having more than one sexual partner, should receive two doses of Imvamune administered by subcutaneous injection at least 28 days apart.

## Introduction

Mpox (previously known as monkeypox) is a viral disease that is typically mild and self-limited, but can lead to severe disease in some populations such as young children, pregnant women and pregnant people and immunocompromised individuals. While outbreaks primarily occur in Central and West Africa, where the monkeypox virus (MPXV) is endemic, a global outbreak occurred in 2022 among previously non-endemic countries, including Canada. Among countries previously non-endemic for the disease prior to 2022, mpox has been primarily transmitted via sexual encounters (83.2%) and among men who have sex with men (MSM) (85.3%). Since 2022, the majority of cases in Canada were among males (96.4%) aged 18–44 years (79.4%), with a median age of 34 years. Non-sexual exposure settings included household contacts, large events/parties, tattoo parlours and the workplace ((1,2)). Among cases with known HIV status, 52.1% were living with HIV. Approximately 4.1% of cases reported to the World Health Organization (WHO) were in healthcare workers, most of whom were exposed in community settings (i.e., exposures not related to work) ((1)). Thirty-five mpox cases were reported among cisgender and transgender women and non-binary individuals assigned female sex at birth in the context of a multi-national case series (136 confirmed mpox cases among 15 countries; cases reported between May 11, 2022, and October 4, 2022) ((3)). Data on mpox cases among sex workers remains limited.

Monkeypox virus clades currently circulating in Europe, the United States (US) and Canada belong to clade II, specifically subclade IIb, which is associated with milder illness than clade I ((4)). Historically, clade I infections were not known to be associated with transmission through sexual contact; however, in March 2023, a cluster of sexually transmitted clade I mpox cases was confirmed in the Democratic Republic of the Congo (DRC). The index case was a man from the DRC who reported having multiple sexual encounters in both Europe and the DRC, which led to an additional five PCR-positive MPXV cases ((5)). This finding shows that mpox transmission through sexual contact extends beyond clade IIb and highlights the need for more routine screening in mpox-endemic and non-endemic regions.

In response to the outbreaks in Canada, the National Advisory Committee on Immunization (NACI) released interim guidance on the use of Imvamune® in the context of ongoing mpox outbreaks. NACI guidance was first limited to post-exposure vaccination (June 2022) ((6)), which was later updated to include interim guidance for pre-exposure vaccination for high-risk groups, primarily MSM with certain risk factors (September 2022) ((7)). Though the 2022 mpox outbreak has subsided, mpox remains a public health concern, both in Canada and internationally. In response to stakeholder feedback, NACI reconvened to discuss expanded use of Imvamune in the context of a targeted routine program (e.g., outside the context of an ongoing mpox outbreak). Updated guidance was released in May 2024 and is summarized below.

## Methods

For this interim guidance, NACI reviewed key questions as proposed by the NACI mpox Working Group (WG), including on the burden of disease to be prevented and the population(s) with greatest disease burden, vaccine safety, vaccine efficacy/effectiveness, vaccine supply and other aspects of the overall immunization strategy. Knowledge synthesis was performed by the NACI Secretariat and supervised by the NACI mpox WG. Following critical appraisal of individual studies, summary tables with risk of bias assessments informed by Cochrane ROB 2.0 and ROBINS-I, as appropriate, were prepared. The NACI Secretariat provided the NACI mpox WG an assessment of the body of evidence using an Evidence to Decision framework, and proposed recommendations for WG input.

NACI considered feedback obtained during 2022 deliberations from stakeholder groups representing the communities and groups considered at high risk of mpox exposure. Input was also provided by the Public Health Ethics Consultative Group during a 2022 consultation, the Canadian Immunization Committee (CIC; August 2023) and the Public Health Agency of Canada. Guidance on the use of Imvamune in the context of international travel was developed in collaboration with the Canadian Committee to Advise on Tropical Medicine and Travel (CATMAT). The description of relevant considerations, rationale for specific decisions and knowledge gaps are described. NACI reviewed the available evidence and approved updated guidance on March 26, 2024.

Further information on NACI’s evidence-based methods is available online.

## Results

### Effectiveness against mpox infection

**Figure A1:**
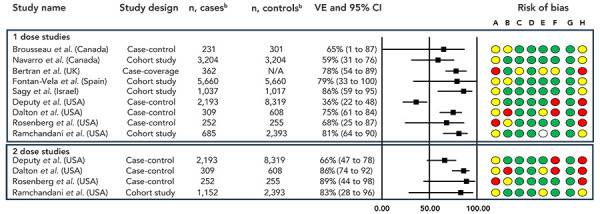
Vaccine effectiveness (and 95% confidence interval) against mpox infection^a,b^ Abbreviations: CI, confidence interval; N/A, not applicable; UK, United Kingdom; USA, United States; VE, vaccine effectiveness ^a^ Studies are stratified by the number of doses administered to participants. A pooled meta-analysis was not performed due to the significant heterogeneity observed across studies. The forest plot depicts estimated vaccine effectiveness (VE) and 95% confidence intervals (CI) of individual studies. Risk of bias legend: A) bias due to confounding; B) bias In selection of participants into the study; C) bias in classification of interventions; D) bias due to deviation from intended interventions; E) bias due to missing data; F) bias in measurement of outcomes; G) bias due to selection of reported result; H) overall risk of bias. Green represented a low risk of bias, yellow a moderate risk of bias, red a serious risk of bias and white represents no information ^b^ Cohort studies are shown as n, vaccinated and n, unvaccinated

Available evidence on the effectiveness of pre-exposure vaccination with Imvamune against mpox was limited to real-world vaccine effectiveness (VE) observational studies. To date, 10 studies have reported estimates of the effect of a single dose of Imvamune against mpox infection ((8–17)), four of which also evaluated the effect of a 2-dose series ((13–16)). One-dose VE against mpox infection ranged from 36% (95% confidence intervals [CI]: 22%–47%) to 86% (95% CI: 59%–95%), while 2-dose VE ranged from 66% (95% CI: 47%–78%) to 89% (95% CI: 44%–98%). All individual studies evaluated are summarized in the **Appendix**, **Figure A1**). Of note, evidence should be interpreted with caution, as studies were assessed to be at a serious risk of bias (largely due to concerns regarding confounding and the measurement of outcomes) or at a moderate risk of bias (Figure A1).

### Effectiveness against moderate/severe mpox infection

**Figure A2:**
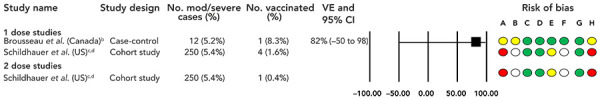
Vaccine effectiveness (and 95% confidence interval) against moderate to severe mpox infection^a,b,c,d^ Abbreviations: CI, confidence interval; mod, moderate; USA, United States; VE, vaccine effectiveness ^a^ The forest plot depicts estimates vaccine effectiveness (VE) and 95% confidence intervals (CI) of individual studies. Risk of bias legend: A) bias due to confounding; B) bias in selection of participants into the study; C) bias in classification of interventions; D) bias due to deviation from intended interventions; E) bias due to missing data; F) bias in measurement of outcomes; G) bias due to selection of reported result; H) overall risk of bias. Green represented a low risk of bias, yellow a moderate risk of bias, red a serious risk of bias and white represents no information ^b^ Define by mpox disease-related hospitalization, having had a complication or having received tecovirimat treatment ^c^ Define as being hospitalized (inpatient hospitalization) for mpox disease ^d^ No VE provided

Two studies provided an estimate of effect of Imvamune against moderate to severe mpox infection. Only Brousseau *et al.* provided an estimate of VE at 82% (95% CI: −50%–98%) for adjusted 1-dose VE. During the study period, 12 individuals had moderate to severe mpox disease, of which three were hospitalized. Only one of these 12 individuals received Imvamune ((8)). A US-based study estimated odds of hospitalization due to mpox among those vaccinated versus those who were unvaccinated. Compared to unvaccinated individuals, the odds of hospitalization among those with mpox who received one or two doses of Jynneos® were 0.27 (95% CI: 0.08–0.65) and 0.20 (95% CI: 0.01–0.90), respectively. Among individuals with both mpox and HIV infections, the odds of hospitalization were 0.28 (95% CI: 0.05–0.91) for those who met the definition of having received one dose of Jynneos, compared to those who were unvaccinated ((17)). Of note, evidence should be interpreted with caution, as studies were assessed to be at a serious risk of bias (largely due to concerns regarding confounding and the measurement of outcomes) or at a moderate risk of bias (Appendix, **Figure A2**).

### Vaccine safety

Both pre- and post-licensure safety data support the safety of Imvamune. According to Imvamune clinical trial data where approximately 13,700 doses were given to 7,414 participants, the most common adverse events reported by adults were injection-site reactions, such as pain, redness and swelling, and systemic reactions, including fatigue, headache and myalgia. Most were mild to moderate in intensity and resolved without intervention within seven days post-vaccination, and no unexpected adverse events were identified. Additionally, there were no confirmed cases of cardiac events such as myocarditis and/or pericarditis following vaccination. The safety profile of Imvamune was similar in both immunocompetent and immunocompromised individuals ((18)).

### NACI recommendations on Imvamune in the context of a focused routine immunization program

**Recommendation 1:** NACI recommends that individuals at high risk of mpox should receive two doses of Imvamune administered at least 28 days (four weeks) apart. **(*Strong NACI recommendation*)**

At this time, individuals considered at high risk of mpox in Canada include:

· Men who have sex with men (MSM)* who meet one or more of the following criteria:

o Have more than one partner; or

o Are in a relationship where at least one of the partners has other sexual partners; or

o Have had a confirmed sexually transmitted infection in the last year; or

o Have engaged in sexual contact in sex-on-premises venues

· Sexual partners of individuals who meet the criteria above

· Sex workers regardless of gender, sex assigned at birth or sexual orientation

· Staff or volunteers in sex-on-premises venues where workers may have contact with fomites potentially contaminated with mpox

· Those who engage in sex tourism regardless of gender, sex assigned at birth or sexual orientation

· Individuals who anticipate experiencing any of the above scenarios

*For the purposes of this NACI guidance, MSM is defined as: Man or Two-Spirit identifying individual who has sex with another person who identifies as a man, including but not limited to individuals who self-identify as transgender, cisgender, Two-Spirit, gender-queer, intersex and non-binary.

**Recommendation 2:** NACI continues to recommend the use of Imvamune as a post-exposure vaccination (also known and referred to as post-exposure prophylaxis) to individuals who have had high risk exposure(s) to a probable or confirmed case of mpox, or within a setting where transmission is happening, if they have not received both doses of pre-exposure vaccination. **(*Strong NACI recommendation*)**

Additional guidance:

· Off-label use in pediatric populations is recommended for those meeting the criteria for post-exposure vaccination and may be offered at their clinician’s discretion.

· Doses should be administered via subcutaneous injection. Dose sparing strategies involving intradermal administration are not recommended in the context of routine immunization.

· At this time, Imvamune is not routinely recommended for healthcare workers, including those serving populations at high risk of mpox, with the exception of post-exposure vaccination.

· Imvamune vaccination can be given concurrently (i.e., same day), or at any time before or after other live or non-live vaccines.

## Conclusion

Due to evolving mpox epidemiology in Canada and emerging evidence on VE of Imvamune, NACI developed national guidance on pre-exposure vaccination in the context of a focused routine immunization program. This included identification of priority populations for pre-exposure vaccination and guidance on a recommended vaccine schedule (summarized below in Appendix, **Table A1**). Note this guidance should be considered interim guidance and will be re-evaluated as additional evidence emerges.

## Appendix

**Table A1:** Immunization schedule for Imvamune® in the context of a focused interim routine immunization program

Dose number	Pre-exposure vaccination^a,b^	Post-exposure vaccination^a,b^
Dose 1	0.5mL, administered via subcutaneous injection (SC)	0.5mL, SC, within 4 days since exposure, can be considered up to 14 days
Dose 2	0.5mL, SC, administered ≥28 days after dose 1	0.5mL, SC, administered ≥28 days after dose 1 if MPXV infection did not develop

Abbreviations: MPXV, monkeypox virus; SC, subcutaneous injection

^a^ Individuals recommended for Imvamune® pre-exposure vaccination should receive a 2-dose schedule regardless of previous vaccination with a live replicating first or second generation smallpox vaccine, immunocompromised status or age

^b^ Pre-exposure or post-exposure vaccination is not indicated for individuals with a history of, or current infection with, MPXV
